# Tenascin-C and Thrombospondin-1 as a Potential Link Between Sleep Bruxism and Cardiovascular Diseases—A Case–Control Study

**DOI:** 10.3390/jcm14186669

**Published:** 2025-09-22

**Authors:** Helena Martynowicz, Monika Kosacka, Piotr Macek, Gabriella Lachowicz, Rafal Poręba, Agnieszka Kusnerz, Aleksandra Jaremków, Cyryl Daroszewski, Agnieszka Bronowicka-Szydełko, Katarzyna Madziarska, Paweł Gać

**Affiliations:** 1 Clinical Department of Diabetology, Hypertension and Internal Diseases, Wroclaw Medical University, Borowska 213 St., 50-556 Wroclaw, Poland; helena.martynowicz@umw.edu.pl (H.M.); gabriela.lachowicz@umw.edu.pl (G.L.); katarzyna.madziarska@umw.edu.pl (K.M.); 2 Department of Pulmonology and Lung Oncology, Wroclaw Medical University, Grabiszynska 105 St., 53-439 Wroclaw, Poland; monika.kosacka@umw.edu.pl (M.K.); cyryl.daroszewski@umw.edu.pl (C.D.); 3 Department of Biological Principles of Physical Activity, Wroclaw University of Health and Sport Sciences, I.J. Paderewskiego St. 35, 51-612 Wrocław, Poland; rafal.poreba@awf.wroc.pl; 4 Department of Population Health, Division of Environmental Health, Occupational Medicine and Epidemiology, Wroclaw Medical University, Mikulicza-Radeckiego St. 7, 50-368 Wroclaw, Poland; agnieszka.kusnerz@umw.edu.pl (A.K.); aleksandra.jaremkow@umw.edu.pl (A.J.); pawel.gac@umw.edu.pl (P.G.); 5 Department of Biochemistry and Immunochemistry, Wroclaw Medical University, Chalubinskiego St. 10, 50-368 Wroclaw, Poland; agnieszka.bronowicka-szydelko@umw.edu.pl

**Keywords:** sleep bruxism, atherosclerosis, tenascin-c, thrombospondin-1, cardiovascular risk

## Abstract

**Background**: Sleep bruxism (SB), a sleep behavior, is one of the most common sleep pathologies. Tenascin-C (TnC) and thrombospondin-1 (TSP-1) are involved in the pathogenesis of cardiovascular disease. The aim of our study was to assess the relationship between SB and TSP-1 and TnC. **Methods**: A total of 80 participants, who were hospitalized in the Sleep Laboratory of the Department of Internal Medicine, Occupational Diseases, Hypertension, and Clinical Oncology at Wroclaw Medical University, were enrolled in the study. Polysomnographic examination was conducted following the standard sleep evaluation criteria set by the American Academy of Sleep Medicine, utilizing the Nox-A1 device. Serum TnC and TSP-1 concentrations were determined using Elisa Kits. **Results**: The study showed an increased concentration of TnC in the group of patients with a BEI (bruxism episode index) >10.3, compared to <10.3 (6786.79 ± 5655.62 vs. 1585.16 ± 2526.56). In regression analysis, higher values of phasic bruxism, smoking, and older age were independently associated with increased concentrations of TnC in the serum. Moreover, higher values of tonic bruxism, as well as smoking, were independently associated with TSP-1. **Conclusions**: Since tenascin-C and thrombospondin-1 levels are associated with sleep bruxism, atherosclerosis may be a potential consequence of SB. This relationship is especially noticeable in severe bruxism. Therefore, monitoring for clinical signs of atherosclerosis should be considered in patients with severe bruxism.

## 1. Introduction

**Figure 1:**
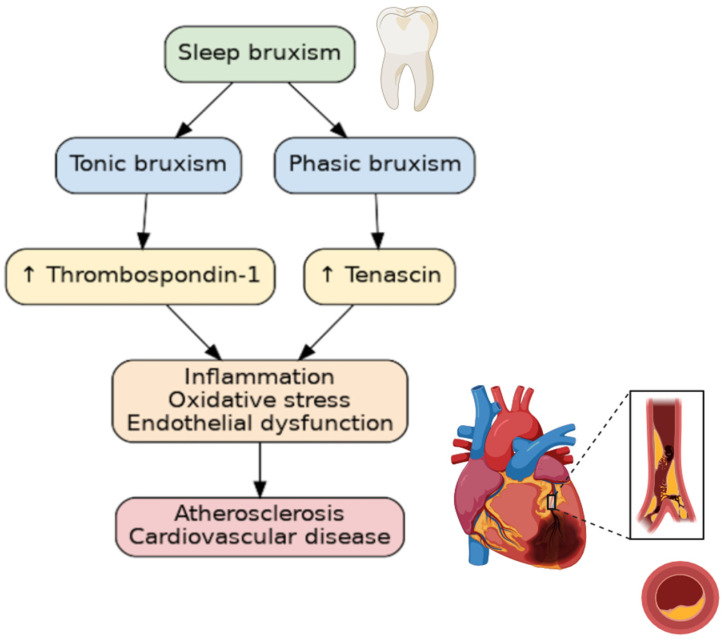
The relationship between bruxism and atherosclerosis. ↑—increasing concentration.

Numerous studies have linked sleep pathologies to cardiovascular diseases. Sleep bruxism (SB), a sleep behavior, is one of the most common sleep pathologies. In fact, SB affects 21% of the world’s population, with the highest prevalence being in North America, at 31%, and the lowest in Asia, at 19% [1]. In 2018, the consensus defined SB as “masticatory muscle activity during sleep”, which is characterized as either being “rhythmic (phasic) or non-rhythmic (tonic)” [2]. SB, which is diagnosed during polysomnographic examination (PSG), occurs predominantly during periods of sleep referred to as non-rapid eye movement (non-REM), and appears to be activated by cardiac or cortical stimulation [3]. SB has long been considered an oral disease, but in recent years, non-dental complications have been described. SB has been associated with elevated blood pressure [4], increased systolic blood pressure variability [5], oxidative stress [6], and inflammatory markers [7]. Furthermore, it has been associated with obstructive sleep apnea, snoring, and a low arousal threshold [8]. Despite SB being an age-old problem, some of the pathomechanisms linking SB to other diseases, particularly atherosclerosis, remain a mystery. Tenascin-C (TnC) and thrombospondin-1 (TSP-1) have both been implicated in the pathogenesis, diagnosis, and prognosis of patients with cardiovascular disease [9,10]. TnC and TSP-1 are two such extracellular matrix (ECM) components that are involved in inflammation, endothelial cell damage, and smooth vascular cell migration. As sleep bruxism is associated with sympathetic overdrive, sleep fragmentation, and increased blood pressure variability, the aim to explore the relationship between SB and factors involved in the pathomechanism of cardiovascular development seems justified (Figure 1).

The direct relationship between SB and the concentration of TSP-1 and TnC in human serum has not yet been studied. The aim of our study was to assess the concentration of TSP-1 and TnC in the serum of individuals diagnosed with sleep bruxism, as well as to investigate the relationship between SB and TSP-1 and TnC. We hypothesize that SB, particularly severe SB, leads to changes in the concentration of TSP-1 and TnC in human serum, thus providing a new explanation for its connection to cardiovascular diseases, and further cementing the relationship between the two.

## 2. Materials and Methods

### 2.1. Study Participants

**Figure 2:**
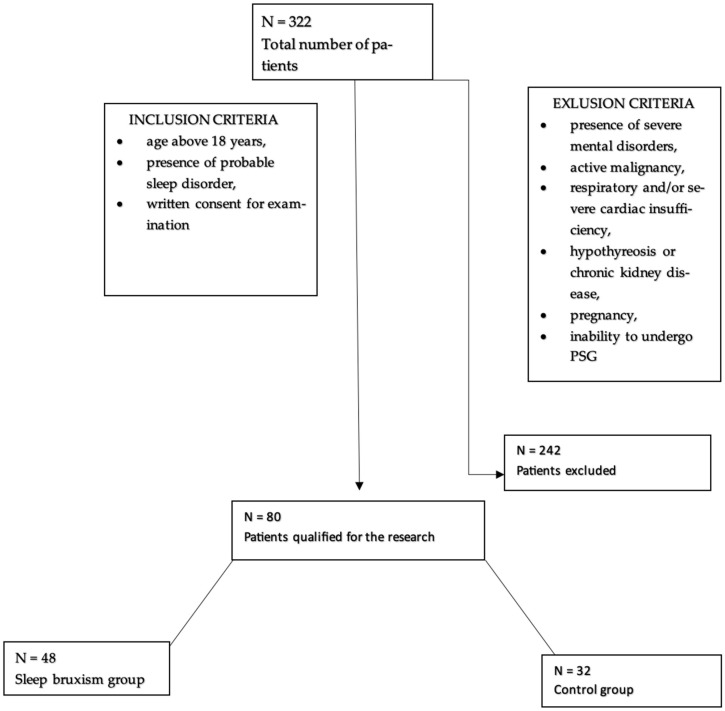
Flowchart for accepting patients into the study.

80 participants, who were hospitalized in the Department of Internal Medicine, Occupational Diseases, Hypertension, and Clinical Oncology at Wroclaw Medical University, for video-PSG, were enrolled in the study. Among them, 55% were men and 45% were women. The participants were divided into two groups, the first of which included 48 participants (60%) with sleep bruxism, while 32 individuals (40%) constituted the control group. The average age was 49.76 ± 16.86. Patients who were included in the study were at least 18 years old, had signed an informed consent form for the study, and had probable sleep disorders. Exclusion criteria included the following: the presence of severe mental illness, cardiac and/or respiratory disease, active inflammation/cancer, other serious metabolic diseases, and pregnancy (Figure 2).

The study was conducted in accordance with the Declaration of Helsinki. Prior to participating in the study, all patients had signed a written informed consent form. The study received approval from the Ethical Committee of Wroclaw Medical University (no. KB-124/2024).

### 2.2. Polysomnography

Polysomnographic examination was conducted according to the standard sleep evaluation criteria set by the American Academy of Sleep Medicine (AASM, 2013) [11], utilizing the Nox-A1 device (Nox Medical, Reykjavík, Iceland). Eye movements were recorded through electrooculography (EOG), while brain bioelectrical activity was recorded using electroencephalography (EEG). Additionally, inductive plethysmography measured chest and abdominal movements, while muscle tension was assessed via electromyography (EMG) from tibial electrodes. Airflow was monitored using a nasal pressure sensor, and the patient’s body position was logged, alongside lateral masseter electromyography (EMC).

After the assessment, a qualified physician from the Sleep Laboratory at Wroclaw Medical University analyzed the gathered data manually in the Noxturnal system (version 2.6). Scoring for sleep disorders was conducted in line with the AASM guidelines.

Standards from the AASM were used to diagnose sleep bruxism (SB). Bilateral EMG recordings were taken from the masseter muscle area, categorizing SB EMG phenotypes as being either phasic, tonic, or mixed. The Bruxism Episode Index (BEI) reflects the total number of SB episodes (phasic, tonic, and mixed) per hour. As per AASM standards, the peak EMG amplitude during a bruxism episode must be at least double that of the background EMG. If the interval between episodes was under 3 s, such intervals were considered to be a part of the same episode. A phasic event was detected if it lasted at least 2 s with three or more pulse patterns, while a tonic episode was identified by sustained pulses lasting longer than 2 s. A mixed episode was recorded when it did not fit the criteria for either a phasic or tonic event. As per the guidelines, SB was diagnosed when the BEI was at least 2, with severity being classified as follows: BEI = 2–4 indicated mild/moderate SB, and BEI > 4 denoted severe SB.

### 2.3. Biochemical Markers

Blood was collected the morning after the polysomnographic examination, usually by puncturing the ulnar vein. It was then stored at a constant temperature until determinations were made simultaneously in all samples.

The Human Tenascin-C, TN-C Elisa Kit (Bioassay Technology Laboratory, Shanghai, China) was used to determine the serum TnC concentration. The determinations were made strictly according to the test manufacturer’s instructions. The TN-c concentration was expressed as nanogram per liter (ng/L). The reference range of the assay used was 20–6000 ng/L. According to the manufacturer, the sensitivity of the ELISA test used was 10.57 ng/L.

The serum TSP-1 level was calculated using the Human Thrombospondin-1, TSP-1 ELISA kit (Bioassay Technology Laboratory, Shanghai, China). These determinations were also made stringently according to the test manufacturer’s instructions. The TSP-1 concentration was expressed as nanogram per milliliter (ng/mL). The reference range of the assay used was 5–700 ng/mL. According to the manufacturer, the sensitivity of the ELISA test used was 2.39 ng/mL.

Apparatus used to read the measurements: PowerWave XS Microplate Spectrophotometer BioTek, Vilnius, Lithuania, absorbance measured at 450 nm. Apparatus used during the procedure: Microplate Washer 50 TS BioTek, Microplate Thermostatic Shaker DTS-2 ELMI Vilnius, Lithuania.

### 2.4. Statistical Analysis

“Dell Statistica 13.1” (Dell Inc., Round Rock, TX, USA) was utilized for the statistical analysis. Arithmetic means and standard deviations (SDs) of the estimated parameters were calculated for quantitative variables. The distribution of these variables was assessed using both the Lilliefors test and the W-Shapiro–Wilk test. For independent quantitative variables, Student’s *t*-test was applied for those with a normal distribution, while the Mann–Whitney U test was used for those with a non-normal distribution. Results for categorical variables were presented as percentages. To explore relationships between the variables studied, correlation and regression analyses were performed. For normally distributed variables, Pearson correlation coefficients (r) were calculated, and Spearman correlation coefficients (r) were used for non-normally distributed variables. The parameters of the model derived from the multivariate regression analysis were estimated using the least squares method. Additionally, the accuracy of the tests was evaluated through an ROC (receiver operating characteristic) analysis. A *p*-value of less than 0.05 (two-tailed) was deemed statistically significant.

## 3. Results

The study included 80 patients, 44 males and 36 females, aged 20 to 81 years. Among the group, 16 (24%) were smokers, 11 (16%) had diabetes, 30 (43%) had arterial hypertension, 6 (9%) had coronary artery disease, 5 (7%) had post-acute coronary syndrome, and 6 (4%) had a history of stroke. Details characterizing the participants are presented in Table 1 and Table 2. The polysomnographic and bruxism parameters that were measured are presented in Table 3.

A comparative analysis was conducted to evaluate the mean concentrations of tenascin-C and thrombospondin-1 between groups with a BEI < 2/h and BEI > 2/h, as well as with BEI > 4 and <4. Detailed data are presented in Table 4.

**Figure 3:**
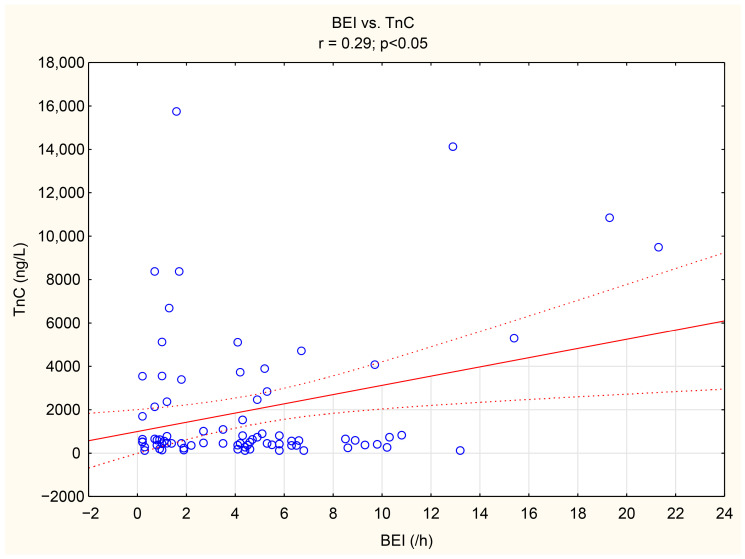
The scatter plot of TnC against BEI.

**Figure 4:**
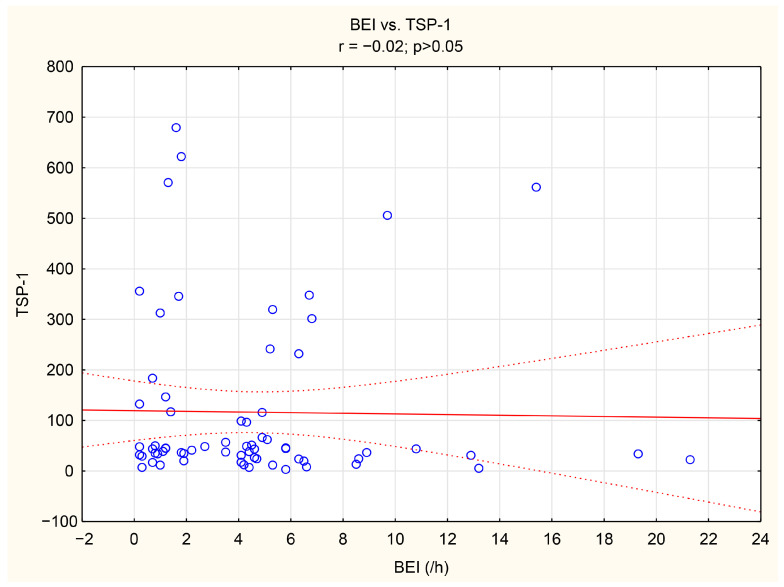
The scatter plot of TSP-1 against BEI.

The associations between tenascin-C, thrombospondin-1, and numerous bruxism parameters were analyzed. TnC exhibited positive correlations with BEI, Phasic bruxism, BEI non-supine, and BEI during the N2 stage of sleep. Similarly, TSP-1 showed a positive correlation with the number of Bruxism Tonic Episodes per hour. Detailed data are presented in Table 5. We have shown the scatter plot of each of TnC and TSP-1 against BEI in Figure 3 and Figure 4.

**Figure 5:**
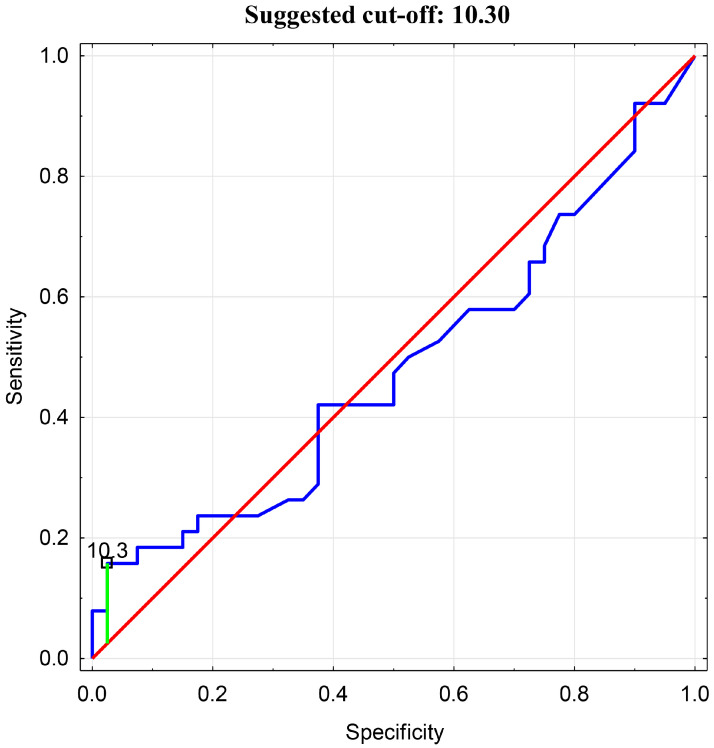
ROC Analysis for BEI cutoff value in relation to Tenascin-C.

Then, an analysis of the receiver operating characteristic (ROC) was performed. An ROC curve analysis and assessment of the predictive accuracy of the test were conducted. The optimal cutoff value for BEI in relation to tenascin-C was determined to be 10.3 (Figure 5).

In the presented analysis, it was determined that a TnC concentration exceeding the median (>601.86) is associated with a BEI > 10.3, with a sensitivity of 54.2%, specificity of 83.3%, and an accuracy of 56.4%. The studied population was then subdivided into groups with either a BEI > 10.3 or <10.3, and a statistically significant difference was found in the comparative analysis (6786.79 ± 5655.62 vs. 1585.16 ± 2526.56, *p* = 0.000).

The regression analysis revealed that TnC levels are significantly associated with phasic bruxism episodes (*p* = 0.035), smoking (*p* = 0.045), and age (*p* = 0.031). Other variables showed no significant correlations. Detailed data are presented in Table 6.

The analysis demonstrated significant associations between TSP-1 levels and smoking (*p* = 0.035) as well as tonic bruxism episodes (*p* = 0.036), indicating that these factors may increase TSP-1 levels in the serum. Other variables showed no significant associations. This suggests that TSP-1 was primarily influenced by lifestyle factors and bruxism activity in this sample. Detailed data are presented in Table 7.

## 4. Discussion

### 4.1. Sleep Bruxism and Its Relationship to Cardiovascular Diseases

Sleep bruxism is a common sleep-related behavior that is usually considered a dental problem leading to tooth wear, temporomandibular disorders, muscle pain, and headaches. However, recent studies indicate that SB may be a possible sleep disruptor involved in sleep fragmentation, and as a result, is followed by cardiovascular complications. Sleep bruxism leads to sleep fragmentation [12], which has recently been considered to be a new cardiovascular risk factor, especially in women [13]. The precise relationship between bruxism and cardiovascular diseases is however, still not clear. Numerous pathomechanisms, such as increased sympathetic activity, systemic inflammation, oxidative stress, endothelial dysfunction, and hormonal changes, can lead to hypertension, heart rate variability, and other cardiovascular complications in sleep bruxers [14]. Sleep bruxism is also associated with blood pressure changes during sleep and leads to an overall rise in arterial blood pressure [4]. Our study indicates that there is a relationship between sleep bruxism and proteins involved in atherogenesis, such as TSP-1 and TnC. The most important result of the study is the independent association between phasic bruxism and the TnC concentration, as well as between tonic bruxism and TSP-1.

### 4.2. The Role of Trombospondin-1 and Tenascin-C in Atherogenesis

TSP-1, which is one of the glycoproteins present in the extracellular matrix (ECM), participates in numerous processes in the body. Studies in vitro showed that TSP-1 inhibits lymphangiogenesis via activation of CD47 in Lymphatic Endothelial Cells (LEC), which suggests that LEC CD47 could, in the future, be a potential therapeutic target in the treatment of atherosclerosis [15]. Clinical studies also support the association between TSP-1 and atherosclerosis. Lin et al. demonstrated that carotid intima-media thickness was greater in hypertensive patients with an increased TSP-1 concentration in the serum, but not in the normotensive group of patients. This result may suggest that TSP-1 could even be a crucial marker of increased susceptibility to atherosclerosis in patients with hypertension [16]. Increased TSP-1 levels have also been described in patients with coronary artery disease and in patients with diabetes mellitus [17]. In the hemodialysis population, a higher TSP-1 concentration is related to cardiovascular disease as well as all-cause and cardiovascular mortality [18]. A meta-analysis study has confirmed the relationship between an increased TSP-1 concentration and cardiovascular complications in diabetic patients. Moreover, it reported a positive correlation between TSP-1 and HbA1c [19].

Numerous studies have demonstrated that tenascin-c plays an important role in the progression of atherosclerosis. Animal studies have shown a link between TnC and many heart diseases, such as myocardial infarction, hypertensive fibrosis, myocarditis and cardiomyopathy [20]. TnC stimulates cardiac fibrosis by accelerating macrophage migration and production of proinflammatory and profibrotic molecules via αVβ3/nuclear factor-κB/interleukin-6 axis [21]. In a few studies, higher TnC serum levels were observed in patients with coronary artery disease, as opposed to patients in the control group [22,23]. Additional observations in the group of patients who had an acute coronary event in the past suggest that TnC may play a crucial role in coronary plaque formation and in the pathogenesis of coronary lesions. Furthermore, some authors propose that TnC could be a useful marker for predicting the severity of an acute coronary syndrome [24]. Other researchers have found correlations between TnC levels and the Gensini Score—an angiographic scoring system—and have suggested that the concentration of TnC in the serum may be helpful in the risk assessment for coronary artery disease, prior to conducting angiography [22]. Li et al. showed that in patients with type 2 diabetes, the serum TnC levels were significantly higher in the group with pre-existing cardiovascular disease. In addition, these authors observed a correlation between TnC and age, waist circumference, and waist/hip ratio [25]. Considering that TnC is overexpressed in atherosclerotic plaques but not in the majority of normal adult tissues, newer studies have concentrated on the possibility that TnC may be a promising delivery vector for anti-atherosclerotic medicines [9].

TnC and TSP-1 may also interact with lipoproteins. The migration of monocytes into the intima and the conversion into foam cells is promoted by oxidized LDL (oxLDL) or acetyl LDL (AcLDL)n and saturated free fatty acids [26]. TnC was shown to be involved in foam cell formation through Toll-like receptor-4. Tenascin-C was produced by oxidized LDL-stimulated macrophages [27]. Thus, Tnc and lipids cooperate in the formation of atherosclerotic plaques. Thrombospondin-1 (TSP-1) is a matricellular extracellular matrix protein. Studies in ApoE−/− mice show that the deficiency of TSP-1 results in more advanced plaques with a larger necrotic core, increased macrophage infiltration, and accelerated plaque progression [28]. In animal models, the overexpressed TSP-1 secreted by injured arteries may bind to very-low-density lipoprotein (VLDL), which may promote its incorporation into nascent atherosclerotic plaques, simultaneously delivering VLDL cholesterol into the lesions [29]. Therefore, research on the role of Tnc and TSP in the formation of atherosclerotic plaques in patients with sleep disorders seems to be a new and interesting field of research.

### 4.3. The Potential Pathomechanisms of Atherosclerosis in Sleep Bruxism

The pathomechanisms linking SB with its cardiovascular complications remain complex and unclear. The dysregulation of the autonomic nervous system during episodes of SB appears to be one of the main culprits linking the two [14]. Stimulation of the sympathetic nervous system dominates and ultimately results in the propagation of the inflammatory process [14], oxidative stress [6], endothelial restructuring [4], hormonal dysfunction, and an increase in the variability of the heart rate, all of which contribute to hypertension, atherosclerotic disease, and other cardiovascular problems. The continued interest displayed by the medical community with regard to atherosclerotic disease is unsurprising, as ischemic heart disease continues to be the most common cause of death worldwide [30]. One angle that is being investigated by researchers is the relationship between atherosclerotic diseases and the concentration of various components in the ECM. At physiologic levels, these components are responsible for maintaining the structure, continuity, and appropriate functioning of the cardiac tissue and blood vessels. In turn, they help modulate diastolic stiffness, manage the repair and remodeling of damaged cardiovascular tissues, provide physical support for cell adhesion, and help regulate both the growth and death of cells [31]. However, disturbances in the concentration of these components may contribute to the formation of atherosclerosis, which is characterized by thickened arterial walls with decreased elasticity due to the formation of plaques [32]. Several processes are considered to form the basis for atherogenesis: inflammation, endothelial cell damage, lipid levels, and vascular smooth muscle cell (VSMC) migration. TnC and TSP-1 are two such ECM components that impact each of these processes. Perhaps the most crucial feature of these proteins is that their concentrations appear to be increased at sites of intimal hyperplasia [33,34]. Both of these glycoproteins seem to play a key role in stimulating the migration of vascular smooth muscle cells (VSMCs) from the tunica media to the tunica intima. On top of that, TSP-1 has been found to further compound already existing pathological processes in the arteries [31]. The glycoprotein is present in high concentrations within the vascular wall, particularly after endothelial damage, which is conducive to the penetration of the wall by monocytes and macrophages, further exacerbating the inflammatory state [35].

In the present study, multivariate logistic regression analysis demonstrated the relationship between tonic bruxism and the concentration of TSP-1. We also showed that smoking is independently associated with TSP-1. To the best of our knowledge, this association, as well as the association between TnC and phasic bruxism, has been demonstrated for the very first time in this study. SB and phasic bruxism correlated with TnC in a statistically significant manner (r = 0.29, *p* < 0.05, r = 0.32, *p* < 0.05, respectively). Moreover, we showed a statistically significant difference in TnC concentration between groups of patients with a BEI < 10.5 vs. >10.3. As a BEI > 4 indicates a severe form of bruxism, the results reveal an increased risk of cardiovascular complications in patients with a very severe form of SB (when the BEI > 10.3).

### 4.4. The Role of Electromyographic Phenotypes of Sleep Bruxism

It is worth noting that we demonstrated different pathways in the possible atherosclerotic process linked to phasic bruxism and tonic bruxism. The results indicate various mechanisms contributing to cardiovascular complications in the tonic and phasic phenotypes of SB. Previous studies have demonstrated that different pathogenic processes are responsible for tonic events, compared to phasic episodes [36,37]; thus, the results are in agreement with these studies. It is worth noting that phasic but not tonic bruxism was reported to be associated with snoring previously [38].

We have also established that smoking is independently related to TnC and TSP-1 levels, indicating the role of proteins in atherogenesis caused by smoking.

### 4.5. Limitations and Strengths

The lack of an adaptive night may be considered a limitation in this study. Regrettably, it was not possible to conduct a night of adaptation due to the organization of the local health service. The sample size (*n* = 80) is not large, and more inflammatory markers need to be determined. However, the strength of the study is the conducted polysomnographic examination, which is a gold standard in the diagnosis of sleep bruxism.

### 4.6. Future Research Directions

The results of our study indicate new research directions. Firstly, the causation relationship between SB and cardiovascular diseases needs to be studied. Moreover, the association between TnC, TSP-1, and obstructive sleep apnea is also worth exploring. Finally, other pathways of atherogenesis in SB, including vascular endothelial dysfunction and nitric oxide pathways, deserve investigation.

## 5. Conclusions

Since Tenascin-C and Thrombospondin-1 are associated with sleep bruxism, atherosclerosis, and cardiovascular diseases may be a potential consequence of SB. However, we did not investigate the causal relationship. The above-mentioned effect is most noticeably observed in bruxism when the BEI is >10.3. Since severe bruxism occurs >4 times per hour, the study results indicate a possible relationship between TnC and TSP-1 and SB in cases of very severe bruxism. Moreover, we demonstrated different pathways in the possible atherosclerotic process linked to phasic bruxism and tonic bruxism. Therefore, further studies are required to explore this association.

## Figures and Tables

**Table 1:** Characteristics of the group.

Parameter	Mean	Minimum	Maximum	SD
Age (years)	49.76	20	81	16.86
Glucose (mg/dL)	104.87	71	216	27.94
Creatinine (mg/dL)	0.93	0.65	1.52	0.14
Uric Acid (mg/dL)	5.22	2.3	11.1	1.47
CRP (C-Reactive Protein) (mg/L)	2.59	0.1	30.4	4.32
ESR (erythrocyte sedimentation rate) (mm/h)	8.06	1	38	7.08
Total Cholesterol (mg/dL)	197.99	105	304	45
HDL (high-density lipoprotein) (mg/dL)	56.33	32	93	14.27
LDL (low-Density Lipoprotein) (mg/dL)	113.18	41	207	39.43
Triglycerides—TG (mg/dL)	137.68	47	472	71.35
Sodium—Na (mmol/L)	139.91	135	149	2.11
Potassium—K (mmol/L)	4.28	3.37	5.17	0.31
Calcium—Ca (mg/dL)	9.35	8.5	10.2	0.3
Magnesium—Mg (mg/dL)	1.93	1.4	2.4	0.21
Iron (µg/dL)	115.7	19	206	48.06
TIBC (Total Iron Binding Capacity) (µg/dL)	339.64	270	482	47.73
Transferrin (g/L)	2.61	1.91	3.95	0.43
Ferritin (ng/mL)	138.61	0.8	452.4	112.52
Vitamin D (ng/mL)	31.71	11.5	132	16.12

**Table 2:** Characteristics of the group.

Parameter	Class	Number	%
Smoking	NO	52	76.47
	YES	16	23.53
Arterial	NO	39	56.52
	YES	30	43.48
CNS disease	NO	63	91.3
	YES	6	8.7
Myocardial infarction	NO	64	92.75
	YES	5	7.25
Stroke	NO	66	95.65
	YES	3	4.35
Diabetes	NO	58	84.06
	YES	11	15.94

**Table 3:** Polysomnographic and bruxism parameters in the studied group.

Parameter	N	Mean	Minimum	Maximum	SD
AHI (n/h)	80	21.99	0.20	97.80	24.58
SL (min)	80	18.90	0.30	148.70	24.35
WASO (min)	80	70.05	0.00	287.00	60.60
SE (%)	80	81.08	45.70	99.00	13.28
N1 (% of TST)	80	7.89	0.20	41.00	7.41
N2 (% of TST)	80	47.68	17.60	186.50	19.33
N3 (% of TST)	80	28.13	0.10	289.50	30.96
REM (% of TST)	80	21.46	0.00	46.70	8.49
Snore (% of TST)	80	21.90	0.00	79.30	21.73
PLMS Index (n/h)	80	8.76	0.00	64.10	14.14
AI (n/h)	80	21.46	0.00	46.70	8.49
RERAs (n/h)	80	0.28	0.00	2.10	0.47
ODI (n/h)	80	19.89	0.50	92.20	22.48
Mean SpO2 (%)	80	81.23	51.00	94.00	9.77
SpO2 Duration < 90%	80	11.85	0.00	70.60	18.13
BEI (n/h)	78	4.63	0.20	21.30	4.31
Phasic BEI (n/h)	78	2.40	0.00	16.10	2.87
Tonic BEI (n/h)	78	1.49	0.00	7.30	1.52
Mixed BEI (n/h)	78	0.74	0.00	4.00	0.83
BEI supine (n/h)	78	8.06	0.00	93.90	12.81
BEI non-supine (n/h)	73	3.14	0.00	21.00	3.82
BEI N1 (n/h)	78	18.25	0.00	75.80	17.61
BEI N2 (n/h)	78	4.84	0.00	26.20	5.24
BEI N3 (n/h)	78	1.88	0.00	10.30	2.31
BEI REM (n/h)	78	4.28	0.00	101.00	11.49

AHI, apnea–hypopnea index; SL, sleep latency; WASO, wake after sleep onset; SE, sleep efficiency; TST, total sleep time (min); N1, sleep stage 1; N2, sleep stage 2; N3, sleep stage 3; REM, rapid eye movement sleep stage; PLMS, periodic limb movements in sleep; AI, apnea index; RERA, respiratory effort-related arousal; ODI, oxygen desaturation index; mean SpO2, mean oxygen saturation; SpO2 Duration < 90%, time (% of TST) with oxygen saturation < 90%; BEI, bruxism episode index.

**Table 4:** Mean concentrations of biomarkers between groups of patients with a Bruxism Episodes Index (BEI) > 2 and those with a BEI < 2, as well as in those with a BEI > 4 and BEI < 4.

Parameter	BEI < 2	BEI > 2	SD < 2	SD > 2	*p*-Value
Tenascin-C	2310.18	3491.42	1782.22	2933.15	0.47
Thrombospondin-1	153.74	201.09	92.85	133.93	0.14
Parameter	BEI < 4	BEI > 4	SD < 4	SD > 4	*p*-value
Tenascin-C	2077.16	3278.25	1910.49	3074.79	0.82
Thrombospondin-1	139.39	190.40	97.90	140.19	0.31

BEI, bruxism episode index; SD, standard deviation.

**Table 5:** The correlations between bruxism activity and concentrations of tenascin-C and thrombospondin-1. Statistically significant correlations are indicated by * (*p* < 0.05).

	Tenascin-C	Thrombospondin-1
BEI (n/h)	0.29 *	−0.02
Phasic BEI (n/h)	0.32 *	−0.07
Tonic BEI (n/h)	0.11	0.04 *
Mixed BEI (n/h)	0.20	0.07
BEI supine	0.14	0.01
BEI non-supine	0.28 *	0.06
BEI N1 (n/h)	0.11	0.06
BEI N2 (n/h)	0.28 *	0.03
BEI N3 (n/h)	0.11	0.06
BEI REM (n/h)	−0.04	−0.06

BEI, bruxism episode index; N1, sleep stage 1; N2, sleep stage 2; N3, sleep stage 3; REM, rapid eye movement sleep stage.

**Table 6:** Regression summary. Dependent variable: Tenascin-C. Statistically significant variables are indicated by boldface (*p* < 0.05).

Variable	b	SE	t(26)	*p*
**Phasic BEI (n/h)**	**807.100**	**362.396**	**2.227**	**0.035**
**Smoking**	**2188.872**	**1078.257**	**1.712**	**0.045**
**Age (year)**	**56.880**	**236.457**	**1.560**	**0.031**
AHI (n/h)	−41.802	28.879	−1.447	0.160
Myocardial Infarction	−3635.262	2939.511	−1.237	0.227
BEI non-supine (n/h)	−286.545	253.524	−1.130	0.269
Heart Disease	2424.893	2788.043	0.870	0.392
Glucose (mg/dL)	−10,935	22,803	−0.480	0.636
BEI N2 (n/h)	−117.857	308.779	−0.382	0.706
Male	430.016	1246.457	0.345	0.733
Stroke	955.630	2876.271	0.332	0.742
BEI (n/h)	90.718	395.028	0.230	0.820
Diabetes	−481.620	2145.273	−0.225	0.824
HDL (mg/dL)	−291.825	1890.910	−0.154	0.879
LDL (mg/dL)	−265.831	1896.151	−0.140	0.890
Triglycerides (mg/dL)	−52.689	379.028	−0.139	0.891
Total Cholesterol level (mg/dL)	263.395	1895.443	0.139	0.891
CRP (mg/L)	13.508	99.595	0.136	0.893
Arterial hypertension	−131.315	1026.467	−0.128	0.899

BEI, bruxism episode index; N2, sleep stage 2; AHI, apnea–hypopnea index; HDL, high-density lipoprotein; LDL, low-density lipoprotein; CRP, C-reactive protein; b, intercept; SE, statistical error.

**Table 7:** Regression summary. Dependent variable: Thrombospondin-1. Statistically significant associations are indicated by boldface.

Variable	b	SE	t(21)	*p*-Value
Smoking	**57.602**	**26.658**	**0.665**	**0.035**
Tonic BEI (n/h)	**5.235**	**2.681**	**0.541**	**0.036**
Arterial hypertension	−94.776	76.253	−1.243	0.228
HDL (mg/dL)	140.228	144.811	0.968	0.344
LDL (mg/dL)	137.680	145.481	0.946	0.355
Total cholesterol level (mg/dL)	−137.581	145.461	−0.946	0.355
Triglycerides (mg/dL)	27.083	28.907	0.937	0.359
AHI (n/h)	−2.401	2.568	−0.935	0.361
Glucose level (mg/dL)	1.548	2.024	0.765	0.453
Age (year)	2.565	3.407	0.753	0.460
Male	68.533	95.774	0.716	0.482
Diabetes	−92.750	212.178	−0.437	0.666
Stroke	−72.635	283.022	−0.257	0.800
CRP (mg/L)	−6.248	28.580	−0.219	0.829
Myocardial Infarction	−21.713	225.135	−0.096	0.924
Coronary Heart Disease	17.392	215.968	0.081	0.937

BEI, bruxism episode index; AHI, apnea–hypopnea index; HDL, high-density lipoprotein; LDL, low-density lipoprotein; CRP, C-reactive protein; b, intercept; SE, statistical error.

## Data Availability

The data presented in this study are available upon request from the corresponding author. The data are not publicly available.
